# Is miR-21 A Therapeutic Target in Cardiovascular Disease?

**DOI:** 10.53941/ijddp.0201003

**Published:** 2023-01-11

**Authors:** Antoinette Holland, Molly Enrick, Arianna Diaz, Liya Yin

**Affiliations:** Department of Integrative Medical Sciences, Northeast Ohio Medical University, Ohio 44272, USA.

**Keywords:** microRNA, miR-21, miR mimic, miR inhibitor, exosomes, heart failure, myocardial infarction, atherosclerosis

## Abstract

microRNA-21 (miR-21) serves a multitude of functions at the molecular level through its regulation of messenger RNA. Previous research has sparked interest in the role of miR-21 as a potential therapeutic target in cardiovascular diseases. miR-21 expression contributes to the differentiation, proliferation, and maturation of many cell types, such as fibroblasts, endothelial cells, cardiomyocytes, and endothelial progenitor cells. The function of miR-21 depends upon its expression level in the specific cell types and downstream targets, which determine cell fate. Under pathological conditions, the expression level of miR-21 is altered, leading to abnormal gene regulation of downstream signaling and cardiovascular diseases such as hypertension, cardiac hypertrophy and fibrosis, atherosclerosis, and heart failure. Agomirs or antagomirs can be introduced into the respective tissue type to reverse or stop the progression of the disease. Exosomes in the extracellular vesicles, which mediate many cellular events with high biocompatibility, have a high potential of efficiently delivering miR-21 to their targeted cells. The critical role of miR-21 in cardiovascular disease (CVD) is indisputable, but there are controversial reports on the function of miR-21 in the same disease. This discrepancy sparks interest in better understanding the role of miR-21 in different tissues under different stages of various diseases and the mechanism of how miR-21 inhibitors work.

## 1. Introduction

Cardiovascular disease (CVD) is the top cause of death and disability worldwide. According to the World Health Organization (WHO), about 17.9 million people died from CVDs in 2019, 32% of all global deaths. In the United States, one person dies from CVD every 34 seconds, and in 2020, about 697,000 people in the United States died from heart disease–20% of total deaths [1]. Moreover, the cost of heart disease has become a financial burden in the US, totaling about $229 billion annually between 2017 and 2018, according to CDC. With the aging of the population and dramatic increases in the prevalence of cardiovascular risk factors such as obesity and diabetes, CVD will continue to be a significant health concern well into the 21st century.

MicroRNAs (miRNA) are a large class of non-coding single-stranded RNA molecules composed of 22 nucleotides that regulate cellular processes via degeneration or regulation of targeted mRNA. miRNAs are made via non-coding regions along the DNA strand, and miRNAs negatively regulate gene expression to control cellular function and some developmental processes. miRNA interacts with the 3’ UTR of their target mRNAs, but it also interacts with other regions, including the 5’ UTR, promotors, and coding sequence [2]. Intragenic miRNAs are produced from introns that help control cellular processes by regulating mRNA, and their transcription is regulated by their own promotor regions independently of a host gene [2]. This biogenesis pathway is a major pathway of interest in studies for the potential use of miRNA in treating CVD. miRNAs play essential roles in development of CVDs and are potential biomarkers and therapeutic targets.

Among the hundreds of miRNAs, microRNA-21(miR-21) has been shown to be involved in myriad pathways related to CVD [3]. miR-21 is essential for maintaining homeostasis in the cardiovascular system via the regulation of vascular smooth muscles cells (VSMC), cardiomyocytes (CM), fibroblast cells, and endothelial cells (EC) [4]. miR-21 is an independently expressed miRNA with its own unique promotor region. The gene encoding the primary transcript for miR-21 locates on chromosome 17q23.2. The pri-miR-21 locates in the intronic region of a gene named TMEM49, and its own promoter independently transcribes it. The expression of miR-21 is regulated by many transcription factors, such as activation protein 1 (AP-1), ETS-family transcription factor (Ets/PU.1), CCAAT enhancer binding protein alpha (C/EBPα), nuclear factor I (NFI), serum response factor (SRF), tumor protein P53 (p53), and signal transducer and activator of transcription 3 (STAT3) [5]. miR-21 is regulated not only during transcription but also post-transcriptionally. For example, transforming cell growth factor beta (TGF-β) and bone morphogentic protein 4 (BMP4) cause upregulation of pre-miR-21, while there is no effect on the primary miR-21 transcript. Drosha further processes primary miR-21 into pre-miR-21, mediated by SMAs [5].

There are many elegant studies and reviews on the magic and mystery of miR-21 [3-11]. We regret if we missed any of them due to the limited length of this paper. The critical role of miR-21 in CVDs as well as the potential therapeutic properties of miR-21 are indisputable. However, there is controversy regarding the results of studies of miR-21 in CVDs. This discrepancy sparks interest in better understanding the function of miR-21 in different tissues under different stages of various diseases and the mechanism of how miR-21 inhibitors work. In this review, we will not repeat the points that have been reviewed and discussed; instead, we will address the role of miR-21 in major CVDs and related discrepancies/caveats as well as potentials for adopting miR-21 mimics or inhibitors as a therapeutic approach in treating patients with CVD.

## 2. The Function of miR-21 in CVD

miR-21 plays a role in the pathogenesis of most, if not all, cardiovascular diseases [9]. Continual interest in the multiple functions of miR-21 regarding its various effects depends upon its downstream targets. Under pathological conditions, the miR-21 expression is either up- or down-regulated in the corresponding cell types. miR-21 has multiple functions in the cardiovascular system, including VSMC proliferation, migration, and survival, endothelial epithelial to mesenchymal transition, and angiogenesis dependent upon its expression in a specific tissue type.

### 2.1. The Function of miR-21 in Atherosclerosis

Atherosclerosis is a chronic and progressive vascular disease with lipid accumulation and inflammation in the arterial walls that can progress in severity leading to a detrimental CVD. miR-21 is abnormally expressed in various cells throughout the cardiovascular system, causing atherosclerosis progression.

#### 2.1.1. miR-21 in the Circulation

In a study using human peripheral blood samples, Telkoparan-Akillilar P et al. showed that compared to healthy controls, miR-21 was downregulated in the circulation of atherosclerosis patients with stable angina who had severe stenosis (at least 70% clogged) [10].

#### 2.1.2. miR-21 in Macrophages

Canfran-Duque A et al. reported that miR-21 was the most abundant miRNA in macrophages. Their bone marrow transplant study demonstrated that Ldlr^−/−^ mice receiving miR-21-deficient bone marrow cells (BMC) developed larger lesions than those receiving wild-type BMC. These results suggest that the ablation of miR-21 is pro-inflammatory and anti-resolution, and miR-21 is vital in the resolution of inflammation. miR-21 limits the progression and promotes the regression of atherosclerosis [12]. However, the caveat of this study is that the lethal dosage of irradiation changed the innate immunity of Ldlr^−−^ mice, and it is unclear if the miR-21 expression changed in vascular EC and SMC after irradiation. A double knockout of miR-21 and Ldlr might better address the role of miR-21 in the development of atherosclerosis.

#### 2.1.3. miR-21 in Smooth Muscle Cells

Upon injury from various vascular diseases, miR-21 is abnormally regulated and causes VSMC dedifferentiation, proliferation, and migration [7,13]. Sun P et al. used a miR-21 knockdown rat to study the effects of miR-21 in atherosclerosis and concluded that miR-21 promotes VSMC proliferation and migration through the Akt/extracellular signal-regulated kinase (ERK) pathway and aggravates atherosclerosis. However, there was no genetic manipulation (knockout/knockdown) of Ldlr or ApoE in the miR-21 knockdown rats, two genes that are commonly knocked out in mouse models of atherosclerosis [13]. Thus, the role of miR-21 in atherosclerosis is uncertain when the animal model of atherosclerosis is not typical.

In another study, miR-21 was upregulated in a rat model of balloon-injured carotid arteries, leading to neointimal growth. Downregulation of overexpressed miR-21 ameliorated neointima formation in injured carotid arteries after angioplasty by reducing cell proliferation and increasing cell apoptosis through PTEN and Bcl-2 pathways. The results suggest that miR-21 is a novel regulatory RNA for neointimal lesion formation [13,14].

#### 2.1.4. miR-21 in Endothelial Cells

miR-21 is important in EC proliferation, migration, differentiation, and inflammatory response for the homeostasis of endothelium under the dynamic changes in the blood vessel. While high-shear stress-induced miR-21 expression change is atheroprotective, low-shear stress-induced miR-21 expression change leads to a pathological EC phenotype [15]. Oscillatory shear stress and low blood flow reduce the proliferation and protection of ECs, making these regions more prone to the development of arteriosclerosis. Thus, miR-21 affects ECs differently upon high versus low shear stress [16]. On the other hand, laminar stress causes the development of atherosclerotic plaque formation because of its impact on EC expression of miR-21. Oscillatory shear stress (OSS) causes an increase in miR-21 expression in EC, which then inhibits its downstream target peroxisome proliferative-activated receptor alpha (PPARα) and activates the AP-1 pathway [7, 15]. A reduction in the inhibition of the PPARα/AP-1 pathway increases the inflammatory response, enhancing the transcription of miR-21 [7, 15]. This positive cycle of inflammation and AP-1 activation induces overexpression of miR-21 in EC and could be pro-atherosclerosis [7,15].

The role of miR-21 in ECs has been associated with NO production. Unidirectional shear stress increases the expression of miR-21 in ECs, leading to a reduction in its downstream target PTEN [17]. This inhibition of PTEN leads to a decrease in apoptosis and an increase in nitric oxide (NO) production within EC through the PI3K/Akt/eNOS pathway [18]. miR-21 maintains NO levels within ECs through distant pathways, such as the PTEN/Akt/eNOS pathway, to maintain the homeostasis of ECs [17]. miR-21 expression in ECs reduces endothelial cell proliferation, migration, and angiogenesis [19].

A study in Human umbilical vein endothelial cells (HUVECs) showed that overexpressing miR-21 reduced apoptosis and enhanced eNOS phosphorylation and NO levels. Overexpression of miR-21 decreases EC migration and proliferation through the Rhob pathway [7]. These data suggest an atheroprotective role of miR-21 [8]. miR-21 also negatively regulates angiogenesis through the direct binding to Rhob, reducing its levels. Celine, Sabatel, et al. showed that increased expression of miR-21 suppressed angiogenesis through a decrease in migration and proliferation but had no effect on apoptosis within EC [20]. However, it is also reported that miR-21 targets PTEN, increases the expression of hypoxia-inducible transcription factor α (HIF-1α) and vascular endothelial growth factor (VEGF), and induces tumor angiogenesis [21]. One note is that the tumor environment is different with leaking vasculature [22].

#### 2.1.5. miR-21 in Endothelial Progenitor Cells (EPCs)

miR-21 in EPCs is important for EC survival upon damage. Peripheral blood levels of patients with atherosclerosis and hypoxic conditions displayed overexpression of miR-21 in EPCs. miR-21 is linked to aging-associated senescence in EPCs in a recent study. Upregulated miR-21 expression in young EPCs causes cell senescence, while downregulation of miR-21 rejuvenates EPCs and enhances EPC angiogenesis in vitro and in vivo [19]. Upregulated miR-21 in EPCs caused a decrease in proliferation, migration, and differentiation of EPC for endothelial repair [20]. Under homeostatic conditions, miR-21’s downstream target WW domain-containing protein 1 (WWP1) directly suppresses transforming growth factor beta (TGF-β) by binding to the 3’UTR region, decreasing the expression of TGF-β [19]. Under pathological conditions, enhanced miR-21 expression led to increased TGF-β signaling and decreased EC proliferation.

### 2.2. The Function of miR-21 in Acute Myocardial Infarction (AMI)

miR-21 expression peaks could be a potential evolutionary biomarker in determining the severity of many CVDs. In patients or animal models with AMI, miR-21 levels are highly expressed in circulation due to ischemia and injury, indicating pathological conditions [7, 23, 24]. This aligns with the report that the enhancement in miR-21 levels depends mainly upon the decrease in the availability of blood flow to the heart during AMI. miR-21 shows potential properties as a new diagnostic approach to confirm the presence of AMI. This approach could help reduce many concerns related to limitations of the pre-existing markers, such as low sensitivity, imprecision, and specific values [23]. Interesting findings showed that miR-21 levels varied depending on the severity of coronary artery stenosis [23]. Abnormal expression of miR-21 in circulation under pathological conditions could be able to determine the progression in severity of AMI.

In a mouse ischemia/reperfusion (IR) model, miR-21 was markedly induced two and seven days post-IR. In the laser-captured infarcted samples, in situ hybridization showed IR-inducible miR-21 located expressed explicitly in the infarct zone of the IR heart, and miR-21 regulated MMP-2 expression in cardiac fibroblasts of by the PTEN pathway [25]. In an AMI rat model, hypoxia caused downregulation of miR-21 expression in the infarcted region of the heart [23,26]. The decreased miR-21 expression in cardiomyocytes led to its target gene programmed cell death 4 (PDCD4) being upregulated, which inhibits its downstream target AP-1, leading to an increase in cardiomyocytes apoptosis [26]. Increased intracellular actions of miR-21 in cardiomyocytes could improve cardiac function post-AMI [7]. Moreover, overexpression of miR-21 by Ad-miR-21 decreased cell apoptosis in the border and infarcted zones of the infarcted rat hearts, suggesting a protective effect of miR-21 against ischemia-induced cardiomyocyte damage. Interestingly, miR-21 has a similar diagnostic ability compared with other markers in AMI, such as Creatine kinase (CK), creatine kinase-MB (CK-MB), and cardiac troponin I (cTnI). This study suggests critical roles for miR-21 in the early phase of acute MI and provides implications for developing new biomarkers [21].

Endothelial rejuvenation plays a considerable role in the improvement after an AMI. A study involving an injection of bone marrow stem cells (BMSC) with miR-21 mimics in AMI animals showed that miR-21 exerted a protective role in activating EC growth via the PTEN/VEGF pathway. Overexpression of miR-21 is protective in the survival of injured ECs by upregulation of EC proliferation along with angiogenesis. However, the BMSCs were injected two weeks after MI, suggests upregulating miR-21 is beneficial in the late stage of MI, not at the early stage [27]. The increase in the expression of miR-21 in the non-infarcted cardiomyocytes serves a protective role for the surrounding fibroblast cells [7]. This increase in miR-21 expression causes fibroblast activation to differentiate into myoblast [28]. Myoblast cells start cell proliferation, migration, and matrix formation to replace the damaged tissue with scar tissue. If this process is not highly regulated, overexpression of miR-21 could lead to cardiac fibrosis, which could further cause heart failure.

### 2.3. The Function of miR-21 in Diabetic Cardiomyopathy

Diabetes mellitus is closely related to obesity as seen throughout many countries [29]. Patients who experience hypertension, coronary artery disease, and many other risk factors are at higher risk of developing diabetic cardiomyopathy, causing abnormal function in the myocardium. Diabetic cardiomyopathy causes an elevation in oxidative stress levels with a systemic inflammatory response that can lead to cardiac fibrosis and contractility dysfunction [30].

In human obesity, miR-21 expression levels are elevated, enhancing adipose tissue proliferation via the modulation of TGF- β signaling. Irregular regulation of adipocyte differentiation by miR-21 pathological expression can cause insulin resistance (IR), increasing the likelihood of developing a hypertensive disorder. Guglielmi V. et al. showed that the level of miR-21 was twice as high in adipocytes of patients with type 2 diabetes (T2D) compared to patients without diabetes [31]. miR-21 levels were overly expressed due to increased serum insulin and glucose levels in T2D patients. Adipose tissue expression of miR-21 led to a further increase in obesity affected by T2D. Under diabetic conditions, miR-21 downregulated the expression of PTEN, enhancing downstream Akt activity [32]. miR-21 is also involved in the deterioration of insulin resistance in the pathological progression from obesity to T2D.

Interestingly, miR-21 responded differently to short- or long-term high glucose treatments in myocardial metabolic flexibility. A study showed miRNA-21 expression in human ventricular cardiac myoblasts AC16 was significantly increased with short-term high glucose treatment, and genes involved in cellular damage increased expression. In contrast, miRNA-21 expression was reduced at seven days of high glucose treatment, damage pathways were activated, and mitochondrial function was compromised. This suggests that the abundance of miRNA-21 in human cardiomyocytes acts as the first defense mechanism against cardiac stress/injury, but its cardioprotective effect relies on the period of insult / injury [33].

Juguilon C et al. reported that miR-21 expression was increased in diabetic mouse coronary ECs, and the ablation of miR-21 prevented the NO-to-H_2_O_2_), switch of the mediator in coronary endothelial-dependent vasodilation in diabetic mice. Cardiac PGC-1α, PPARα, and eNOS were increased, and endothelial superoxide was reduced in miR-21-deficient mice. It suggests that miR-21 plays a regulatory role in the coronary microcirculation in diabetic cardiomyopathy [34].

Li X. et al. showed that miR-21 was significantly upregulated in diabetes mellitus, and miR-21 expression inhibits autophagy via the sprouty RTK Signaling Antagonist 1 (SPRY1)/extracellular regulated protein kinase (ERK)/mammalian target of rapamycin (mTOR) pathway for normal regulation of the heart [35] Inhibiting miR-21 expression in cardiomyocytes showed an increase in SPRY1 level while decreasing levels of p-ERK and p-mTOR. When miR-21 was induced in these cells, the opposite effect occurred, suggesting that miR-21 expression increases the downstream targets ERK and mTOR in degrading dysfunctional properties in the heart.

Though the above studies showed that miR-21 was upregulated in diabetes, Dai B et al. reported that the levels of miR-21 in cardiomyocytes were decreased in diabetic *db/db* mice. In this study, miR-21 expression was elevated or inhibited by injecting adeno-associated virus (AAV) into eight-week-old *db/db* mice via tail vein. They concluded that miR-21 protected against diabetic cardiomyopathy [36]. It is shown that miR-21 had a protective role against cardiac dysfunction and hypertrophy, and increased miR-21 expression reduced reactive oxygen species (ROS) production via gelsolin [36]. miR-21 increased Akt and eNOS while simultaneously inhibiting the activity of its downstream target gelsolin (GSN) [36]. miR-21 overexpression decreased the level of GSN, while miR-21 inhibition increased it. GSN levels were also increased in the cardiac tissue of obese mice. miR-21 induced adipocyte differentiation by regulating ROS and NO levels via the GSN pathway [37]. The results from this study are different from others, but some caveats of this study may explain the discrepancy: (1) The age of the *db/db* mice with lower miR-21 expression is unclear, which is relevant because young *db/db* might not have DCM. (2). It is unclear about the CT value of real-time PCR of miR-21, for which Trizol instead of miRNeasy was used to extract the miRNA. Also, the reagents used to detect miR-21 were different from the ThermoFisher products that have been used for other studies. The efficiency of extracting the miRNA or the sensitivity/ accuracy of measuring miR-21 might be compromised. (3) AAV will change innate immunity. miR-21^−/−^ and *db/db* mice will better address the role of miR-21 in DCM. (4) More parameters of diastolic function such as early to late diastolic transmitral flow velocity (E/A), E to early diastolic mitral annular tissue velocity (E/e'), and isovolumic relaxation time (IVRT) would be helpful. (5) The measurement of NO and ROS were not performed by electron paramagnetic resonance (EPR) spectroscopy or high-Pressure Liquid Chromatography (HPLC), which are more accurate and reliable.

To investigate the therapeutic role of miR-21 in diabetic cardiomyopathy, Seeger T et al. treated aged *db/db* mice with locked nucleic acid-modified anti-miR-21 (LNA-21) for 18 weeks, but cardiac function did not change. LNA-21 treatment resulted in significant weight loss, reduced adipocyte size, and derepressing of TGFRB2, PTEN, and Sprouty1 and 2 [38]. However, caveats of this study are that the results were based on a small sample size (n=5), and LNA −21 was injected into 18-week-old *db/db* mice when the *db/db* mice had already developed diabetic cardiomyopathy. The intervention of miR-21 expression might be too late because there may be a limited time window for the treatment. Also, more parameters of the diastolic cardiac function as listed above would be helpful.

### 2.4. The Function of miR-21 in Hypertension

miR-21 expression was positively correlated with blood pressure in spontaneous hypertension rats (SHR) and hypertensive patients. miR-21 expression was increased in SHR compared to Wistar rats, and circulating miR-21 levels in hypertensive patients were similarly higher than in controls [39]. Overexpression of miR-21 by AAV was sufficient to decrease blood pressure and alleviate cardiac hypertrophy in SHRs by upregulating mitochondrial translocation. Nuclear DNA synthesis was increased, and mitochondrial DNA synthesis was decreased, causing an increase in intracellular ROS. The authors concluded that induced miR-21 was part of the compensatory program [39]. However, the caveat of this study is that some crucial data, such as diastolic pressure, blood vessel activity, and eNOS expression, were not shown. It is unclear which miR-21 expression in which cell type was responsible for the effect of lower hypertension. Also, ROS measurement by EPR or HPLC might be more convincing. There is again a concern about innate immunity change caused by AAV.

### 2.5. The Function of miR-21 in Cardiac Hypertrophy Fibrosis, and Heart Failure

CVDs, including hypertension, ischemic heart disease, and heart failure, can arise from cardiac hypertrophy [40]. The elevated miR-21 expression in cardiomyocytes is shown to cause cardiac hypertrophy. The intensity of the expression of miR-21 in cardiomyocytes contributing to cardiac hypertrophy differs depending on the developmental age of mouse models [4]. Cardiac fibrosis is a pathological condition contributing to heart failure. The miR-21 expression in cardiac fibroblasts is much more abundant than in cardiomyocytes [4]. The preferential expression of miR-21 in nonmyocyte cell types was further upregulated upon various cardiac diseases associated with cardiac fibrosis [41]. miR-21 has been shown as a central regulator of cardiac fibrosis, and studies in both small and big animals showed inhibition of miR-21 had an anti-fibrotic effect. [41,42]. miR-21 regulates many cellular processes of myocardial fibrosis and is predicted as a therapeutic target for heart failure with preserved ejection fraction (HFpEF) [3].

Studies have shown that miR-21 is weakly expressed in cardiac tissue, limiting cell growth and apoptosis via the PTEN pathway. In cardiac fibroblasts, miR-21 maintains normal tissue production by regulating downstream targets SPRY1 and ERK-mitogen activated protein kinase (MAPK) pathway [5]. During heart failure and ischemic conditions, miR-21 is overexpressed in cardiac fibroblasts leading to cardiac fibrosis. Enhanced expression of miR-21 in cardiac fibroblasts showed a decrease in downstream target SPRY1, an anti-fibrotic protein molecule signaling leading to activation of the ERK/MAPK pathway and causing cardiac hypertrophy [42]. PTEN expression in the fibrotic infarct tissue was downregulated, further suggesting its role in the cascade of cellular events in vascular disease of downstream targets of miR-21. Decreased PTEN expression caused a decrease in cellular apoptosis. As the hypertrophic heart worsened, miR-21 levels increased, but as the patient’s disease progressed to heart failure, there was no change in miR-21 levels [7].

Thum T et al. showed that miR-21 expression is increased selectively in fibroblasts in the failing heart, enhancing ERK–MAP kinase activity through inhibition of Spry1. In a mouse model of pressure overload, silencing miR-21 in vivo by delivering a specific antagomir via jugular vein catheter decreases cardiac ERK–MAP kinase activity, inhibits interstitial fibrosis, and ameliorates cardiac dysfunction [42]. The same group also studied it in a large animal model. When antimiR-21 was locally delivered via intracoronary by catheter in a pig model of heart failure, LNA-21 reduced cardiac fibrosis and hypertrophy and improved cardiac function. Deep RNA-sequencing and single-cell RNA-sequencing revealed decreased numbers of macrophages and fibroblasts, two critical cell types affected by antimiR-21 treatment [41]. It further supported their previous studies in mice [42].

In contrast, it was reported that overexpression of miR-21-3p by AAV markedly reduced cardiac hypertrophy induced by TAC via targeting histone deacetylase-8 and also blocked angiotensin 2 (Ang II-induced cardiac hypertrophy [43]. Furthermore, the role of miR-21 in cardiac hypertrophy and fibrosis became more confusing when Patrick DM et al. showed that cardiac stress responses were unperturbed in miR-21 knockout mice [44]. In the same study, acute inhibition of miR-21 through systemic delivery of an 8-nt locked nucleic acid-modified (LNA-modified) antimiR oligonucleotide did not change the pathological cardiac responses to pressure overload or other stresses either [44]. The compensatory mechanisms activated in the persistent deficiency of miR-21 might explain the discrepancy between the Thum and Patrick studies. It is also possible that cholesterol-conjugated chemistry in the Thum study has a cardioprotective effect, or antagomirs might also be more efficient than the LNA-modified antimiRs used in the Patrick study to inhibit miR-21 function in cardiac fibroblasts in vivo [6, 44]. Other potential possibilities could be miRNA redundancy or nonspecific effects of high levels of a cholesterol-modified antagomir on the heart [6]. These results suggest the gaps in our knowledge of the mechanisms of how miRNAs act and how oligonucleotide-based targeting strategies inhibit miRNAs. Moreover, the route to deliver the antimiR-21(tail vein vs. jugular vein) could cause the efficiency of the antimiR-21 to be different. Therefore, it will be essential to include technical details, dosage regimen, and proper controls in the antagomir studies and carefully analyze and interpret the data from them [6].

## 3. The Therapeutic Potential of miR-21

Several studies have shown miRNA’s importance in maintaining the cardiovascular system’s normal physiology. Understanding the critical role of miR-21 as a biomarker and protector and its specific impact on each tissue type is of interest in treating CVD. miR-21 has many potential therapeutic properties in the case of several underlying diseases that could further contribute to CVD.

When considering miRNA as a potential therapy, several important points to consider are that miRNA can be introduced into the body via multiple mechanisms [28]. When miRNA is introduced into circulation, it undergoes many cellular events that could destroy its structure [45]. The mechanism of miR-21 function differs depending on cell type, so when introduced into the body, miRNA must be chemically modified to target the tissue type of interest [11,28]. In CVD, miR-21 expression is either upregulated or downregulated due to the pathological changes within the associated cells. The miR-21 expression can be manipulated by using antagomirs that down-regulate its expression via the downregulation of endogenous RNA [46]. Upon cellular uptake, antagomirs can knock down miRNA significantly and effectively relieve cardiac pathology [42]. To increase the expression of miRNA, synthetic RNA duplexes called agomirs can mimic a particular endogenous miRNA [28,46]. To increase the specificity of either an antagomir or agomir, several processes can further be utilized to modify their structure to gain specificity and stability.

Despite the controversy over whether miR-21 inhibition decreased myocardial hypertrophy and fibrosis in specific disease models, miR-21 still has the potential to be a therapeutic target for the prevention of HFpEF. However, more studies are needed to reexamine miR-21 inhibition by oligonucleotide-based therapeutics in myocardial hypertrophy and fibrosis with more parameters for diastolic cardiac function. More clinical data in human subjects with diastolic dysfunction are required to understand better if inhibition of miR-21 may decrease myocardial hypertrophy and interstitial fibrosis [3].

miR-21 inhibitor Lademirsen has been used in Phase II trials in patients with Alport syndrome (HERA) to improve kidney phenotype and survival (https://clinicaltrials.gov/ct2/show/NCT02855268) [41, 47, 48]. This clinical trial made it promising to target miR-21 as a therapeutic approach in HFpEF and myocardial fibrosis, but details of the strategy for inhibiting miR-21 and the timepoint and stage of the pathology of HFpEF at intervention/ treatment should be considered.

## 4. Therapeutic Potential of Exosome-Based miR-21 Delivery

Current approaches for delivery of miRNAs include hydrodynamic injection, viral vectors, liposomes, and nanocarriers. The adverse effects of these approaches include toxicity, activating an immune response, tumorigenicity, and low delivery efficiency to the target cells [49, 50]. miR-21 has great potential as a therapeutic agent in treating CVD but has several limitations, including poor cellular uptake and inadequate stability in vivo [51]. Exosomes are an emerging therapeutic option for the introduction of miR-21 into injured tissues for CVDs with the advantage that exosomes are biocompatible, highly abundant, non-immunogenic, and plastic [52].

Interestingly, cells actively release miRNAs, and do so mainly in the form of exosomes. Vesicular miRNAs can serve as biomarkers, but they also have therapeutic potential. Extracellular vesicles can mediate cell-cell communication and numerous biological and pathological processes, including cell proliferation and differentiation, immune reaction, angiogenesis, stress response, tissue repair, regeneration, and senescence [52-54]. Exosomes can effectively transport miRNA, mRNA, proteins, and lipids to their target cells without severe adverse effects [55]. Exosomes mediate signal transfer, cell survival and apoptosis, angiogenesis, and cardiovascular repair and regeneration [53,54,56]. Research studies indicate that miRNAs in the exosomes secreted from donor cells modulate the targeted gene expression in the recipient cells [55]. Another attractive potential of exosomes as a therapeutic approach is that the level and expression of miR-21 being exported can be modified via different conditioning techniques or genetic engineering [57]. Exosomes can be sequestered from various cells, including cardiac progenitor cells, embryonic stem cells (ESC), induced pluripotent stem cells (iPSC), mesenchymal stem cells, and bone marrow stem cells, demonstrating beneficial modulatory effects of exosomes on the myocardial-infarcted heart [49,58-60].

In a study using human endometrium-derived mesenchymal stem cells (EnMSCs) to treat rat myocardial infarction, the cardiac protection of EnMSCs was from the paracrine effect mediated by secreted exosomes. miRNA array and quantitative PCR (qPCR) analyses of exosomal miRNAs showed that miR-21 expression was increased. The EnMSCs lost their antiapoptotic and angiogenic capability after anti-miR-21 treatment. The study implicates miR-21 in the exosomes of EnMSC as a potential mediator of stem cell therapy by enhancing cell survival through the PTEN/Akt pathway [61]. Interestingly, miR-21-3p (miR-21*) was identified in fibroblast-derived exosomes by confocal imaging [62]. Inhibition of miR-21* by pharmacological inhibitor ameliorated the cardiac hypertrophy induced by Ang II, suggesting fibroblast-derived miR-21* as a potential therapeutic target in cardiac hypertrophy [62].

Stem cell-derived exosome miRNAs regulate the survival of cardiomyocytes, the function of cardiac progenitors and ECs, angiogenesis, and cardiac remodeling after myocardial infarction [49]. Exosomes carrying miR-21 play an essential role in cardiac regeneration. Qiao L et al. reported that miR-21-5p expression in the exosomes from explant-derived cardiac stromal cells (CSC) from heart failure patients was decreased compared to the exosomes from normal donor hearts. The reduction of miR-21 expression in the exosomes from failing hearts was related to the impaired capability to improve the remodeling of mouse hearts by decreased apoptosis of cardiomyocytes and pro-angiogenesis after acute MI. Moreover, restoring miR-21 expression in the exosomes of failing hearts rescued the regenerative capability of the exosome [56]. However, limitations of the study include the low n number of the sample size and that the exosome was extracted from the cultured medium of explant-derived CSC instead of from blood samples from patients with heart failure. The secretome of tissue explant/cell culture depends on the cell culture conditions and components of the culture medium. It is uncertain whether the miR-21 expression level in the cell culture mimics the expression of miR-21 in a failing heart. But the study clearly shows the presence of miR-21 in exosomes, the capability of exosomes carrying miR-21, and the function of exosomes. Another study showed a local injection of miR-21-enriched extracellular vesicles (EVS) derived from Hek293T cells in the mouse heart restores cardiac function after myocardial infarction [50]. miR-21-EVS remained inactive until proper transport into its target cell, where it localized in the perinuclear compartment of cardiomyocytes and the cytoplasm of EC. Once inside the cardiomyocytes, miR-21-EVS decreased its downstream target PDCD4, inhibiting cellular apoptosis of cardiomyocytes. The concentration of miR-21 in cardiac fibroblasts was also increased, though cardiomyocytes up took much more miR-21 than fibroblasts did. miR21-EVS also promotes angiogenesis [50]. These results demonstrate that exosome-carrying miR-21 and miR21-EVS treatment promoted cardiac functional recovery after acute MI. One note is that the time of delivery of the exosomes from CSC or miR21-EVS was at the same time as the ligation of LAD, which is different from other studies of miR-21 inhibition in MI models. Again, the intervention time in which to regulate miR-21 could affect the outcome of the disease model.

## 5. Conclusion

Even though miR-21 has been studied extensively in CVD, there are still some gaps in our understanding of miRNA-based regulation of gene expression in the normal and diseased cardiovascular system [63]. With the development of synthetic oligonucleotides, therapeutic approaches based on antimiRs or miRNA mimics are feasible. The typical three-week half-lives of miRNA modulators in cardiac tissue allow treatment to produce effect durations of as long as 18 – 46 days in mice or 28 days in pigs [64]. Although several studies have successfully manipulated the expression of miRNA-21 in several diseases, many challenges are still faced. In the potential use of the antisense oligonucleotides, there is still room for improvement to regulate the desired target effects of miR-21. It is neither organ- nor cell-type-specific and functions distinctly depending on the organ or progression of the disease [46].

The function of miR-21 is dependent upon its level of expression and specific tissue, including EC, VSMC, fibroblasts, or cardiomyocytes. Delivering these molecules to specific cardiovascular tissues is still challenging. The route of oligonucleotide delivery might be essential to improve the uptake efficiency [64]. More research is needed to address the gaps in our knowledge of the mechanisms of how miRNA acts and the different roles of miR-21 at different stages of various diseases. More careful consideration is required for oligonucleotide-based targeting strategies for miR-21 inhibition. It will also be essential to include proper controls in the antagomir studies and carefully analyze and interpret their data [6].

## References

[1] Tsao CW; Aday AW; Almarzooq ZI; Heart disease and stroke statistics-2022 update: a report from the American heart association. Circulation, 2022, 145(8): el53–e639. 10.1161/CIR.0000000000001052 35078371

[2] O'Brien J; Hayder H; Zayed Y; Overview of microRNA biogenesis, mechanisms of actions, and circulation. Front. Endocrinol, 2018, 9: 402. 10.3389/fendo.2018.00402 PMC6085463 30123182

[3] Ben-Nun D; Buja LM; Fuentes F Prevention of heart failure with preserved ejection fraction (HFpEF): reexamining microrna-21 inhibition in the era of oligonucleotide-based therapeutics. Cardiovasc. Pathol, 2020, 49: 107243. 32629211 10.1016/j.carpath.2020.107243

[4] Cheng YH; Zhang CX MicroRNA-21 in cardiovascular disease. Journal of Cardiovascular Translational Research, 2010, 3(3): 251–255. 20560046 10.1007/s12265-010-9169-7 PMC3611957

[5] Kumarswamy R; Volkmann I; Thum T Regulation and function of mirna-21 in health and disease. RNA Biology, 2011, 5(5): 706–713. 10.4161/rna.8.5.16154 PMC3256347 21712654

[6] Morrisey EE The magic and mystery of miR-21. J. Clin. Invest, 2010, 120(11): 3817–3819. 20978356 10.1172/JCI44596 PMC2964999

[7] Dai BB; Wang F; Nie X; The cell type-specific functions of mir-21 in cardiovascular diseases. Front. Genet, 2020, 11: 563166. 33329700 10.3389/fgene.2020.563166 PMC7714932

[8] Sekar D; Venugopal B; Sekar P; Role of microRNA 21 in diabetes and associated/related diseases. Gene, 2016, 582(1): 14–18. 26826461 10.1016/j.gene.2016.01.039

[9] Matsumoto T; Hwang PM Resizing the genomic regulation of restenosis. Circ. Res, 2007, 100(11): 1537–1539. 17556665 10.1161/CIRCRESAHA.107.101103

[10] Telkoparan-Akillilar P; Cevik D Identification of miR-17, miR-21, miR-27a, miR-106b and miR-222 as endoplasmic reticulum stress-related potential biomarkers in circulation of patients with atherosclerosis. Mol. Biol. Rep, 2021, 48(4): 3503–3513. 33860430 10.1007/s11033-021-06352-7

[11] Canfrán-Duque A; Rotllan N; Zhang XB; Macrophage deficiency of miR-21 promotes apoptosis, plaque necrosis, and vascular inflammation during atherogenesis. EMBO Mol. Med, 2017, 9(9): 1244–1262. 28674080 10.15252/emmm.201607492 PMC5582411

[12] Sun P; Tang LN; Li GZ; Effects of MiR-21 on the proliferation and migration of vascular smooth muscle cells in rats with atherosclerosis via the Akt/ERK signaling pathway. Eur. Rev. Med. Pharmacol. Sci, 2019, 23(5): 2216–2222. 30915769 10.26355/eurrev_201903_17269

[13] Ji RR; Cheng YH; Yue JM; MicroRNA expression signature and antisense-mediated depletion reveal an essential role of MicroRNA in vascular neointimal lesion formation. Circ. Res, 2007, 100(11): 1579–1588. 17478730 10.1161/CIRCRESAHA.106.141986

[14] Neth P; Nazari-Jahantigh M; Schober A; MicroRNAs in flow-dependent vascular remodelling. Cardiovasc. Res, 2013, 99(2): 294–303. 23612583 10.1093/cvr/cvt096

[15] Silacci P; Formentin K; Bouzourène K; Unidirectional and oscillatory shear stress differentially modulate NOS III gene expression. Nitric Oxide, 2000, 4(1): 47–56. 10733872 10.1006/niox.2000.0271

[16] Kuang DB; Zhou JP; Yu LY; DDAH1-V3 transcript might act as miR-21 sponge to maintain balance of DDAH1-V1 in cultured HUVECs. Nitric Oxide, 2016, 60: 59–68. 27663503 10.1016/j.niox.2016.09.008

[17] Weber M; Baker MB; Moore JP; MiR-21 is induced in endothelial cells by shear stress and modulates apoptosis and eNOS activity. Biochem. Biophys. Res. Commun, 2010, 393(4): 643–648. 20153722 10.1016/j.bbrc.2010.02.045 PMC3717387

[18] Zuo KQ; Li MQ; Zhang XP; MiR-21 suppresses endothelial progenitor cell proliferation by activating the TGFβ signaling pathway via downregulation of WWP1. Int. J. Clin. Exp. Pathol, 2015, 8(1):414–422. 25755729 PMC4348897

[19] Sabatel C; Malvaux L; Bovy N; MicroRNA-21 exhibits antiangiogenic function by targeting RhoB expression in endothelial cells. PLoS One, 2011, 6(2): e16979. 21347332 10.1371/journal.pone.0016979 PMC3037403

[20] Liu LZ; Li CY; Chen Q; MiR-21 induced angiogenesis through AKT and ERK activation and HIF-1α expression. PLoS One, 2011, 6(4): el9139. 10.1371/journal.pone.0019139 PMC3081346 21544242

[21] Kanugula AK; Adapala RK; Jamaiyar A; Endothelial TRPV4 channels prevent tumor growth and metastasis via modulation of tumor angiogenesis and vascular integrity. Angiogenesis, 2021, 24(3): 647–656. 33656628 10.1007/s10456-021-09775-9 PMC8295186

[22] Zhang Y; Liu YJ; Liu T; Plasma microRNA-21 is a potential diagnostic biomarker of acute myocardial infarction. Eur. Rev. Med. Pharmacol. Sci, 2016, 20(2): 323–329. 26875904

[23] Wang F; Long GW; Zhao CX; Atherosclerosis-related circulating mirnas as novel and sensitive predictors for acute myocardial infarction. PLoS One, 2014, 9(9): e105734. 25184815 10.1371/journal.pone.0105734 PMC4153586

[24] Roy S; Khanna S; Hussain SRA; MicroRNA expression in response to murine myocardial infarction: miR-21 regulates fibroblast metalloprotease-2 via phosphatase and tensin homologue. Cardiovasc. Res, 2009, 82(1): 21–29. 19147652 10.1093/cvr/cvp015 PMC2652741

[25] Dong SM; Cheng YH; Yang J; MicroRNA expression signature and the role of microRNA-21 in the early phase of acute myocardial infarction. J. Biol. Chem, 2009, 284(43): 29514–29525. 19706597 10.1074/jbc.M109.027896 PMC2785585

[26] Yang F; Liu WW; Yan XJ; Effects of mir-21 on cardiac microvascular endothelial cells after acute myocardial infarction in rats: role of phosphatase and tensin homolog (PTEN)/vascular endothelial growth factor (VEGF) signal pathway. Med. Sci. Monit, 2016, 22: 3562–3575. 27708252 10.12659/MSM.897773 PMC5056537

[27] Jayawardena E; Medzikovic L; Ruffenach G; Role of mirna-1 and mirna-21 in acute myocardial ischemia-reperfusion injury and their potential as therapeutic strategy. Int. J. Mol. Sci, 2022, 22(3): 1512. 10.3390/ijms23031512 PMC8836257 35163436

[28] Piché ME; Tchernof A; Després JP *Obesity phenotypes*, diabetes, and cardiovascular diseases. Circ. Res, 2020, 126(11): 1477–1500. 32437302 10.1161/CIRCRESAHA.120.316101

[29] Rai AK; Lee B; Gomez R; Current status and potential therapeutic strategies for using non-coding RNA to treat diabetic cardiomyopathy. Front. Physiol, 2021, 11: 612722. 33551838 10.3389/fphys.2020.612722 PMC7862744

[30] Guglielmi V; D'adamo M; Menghini R; Microrna 21 is up-regulated in adipose tissue of obese diabetic subjects. Nutr. Healthy Aging, 2017, 4(2): 141–145. 28447068 10.3233/NHA-160020 PMC5389040

[31] Kantharidis P; Wang B; Carew RM; Diabetes complications: the microRNA perspective. Diabetes, 2011, 60 (7): 1832–1837. 21709278 10.2337/db11-0082 PMC3121430

[32] Scisciola L; Benedetti R; Chianese U; The pivotal role of mirna-21 in myocardial metabolic flexibility in response to short- and long-term high glucose treatment: evidence in human cardiomyocyte cell line. Diabetes Res. Clin. Pract, 2022, 191: 110066. 36058441 10.1016/j.diabres.2022.110066

[33] Juguilon C; Wang ZY; Wang Y; Mechanism of the switch from no to H_2_O_2_ in endothelium-dependent vasodilation in diabetes. Basic Res. Cardiol, 2022, 117(1): 2. 35024970 10.1007/s00395-022-00910-1 PMC8886611

[34] Li XC; Meng C; Han F; Vildagliptin attenuates myocardial dysfunction and restores autophagy via mir-21/spry1/erk in diabetic mice heart. Front. Pharmacol, 2021, 12: 634365. 33815116 10.3389/fphar.2021.634365 PMC8013777

[35] Dai BB; Li HP; Fan JH; Mir-21 protected against diabetic cardiomyopathy induced diastolic dysfunction by targeting gelsolin. Cardiovasc. Diabetol, 2018, 17(1): 123. 30180843 10.1186/s12933-018-0767-z PMC6122727

[36] Tochhawng L; Deng S; Pugalenthi G; Gelsolin-Cu/ZnSOD interaction alters intracellular reactive oxygen species levels to promote cancer cell invasion. Oncotarget, 2016, 7(33): 52832–52848. 27391159 10.18632/oncotarget.10451 PMC5288152

[37] Seeger T; Fischer A; Muhly-Reinholz M; Long-term inhibition of miR-21 leads to reduction of obesity in db/db mice. Obesity, 2014, 22(11): 2352–2360. 25141837 10.1002/oby.20852

[38] Li HP; Zhang XR; Wang F; MicroRNA-21 lowers blood pressure in spontaneous hypertensive rats by upregulating mitochondrial translation. Circulation, 2016, 134(10): 734–751. 27542393 10.1161/CIRCULATIONAHA.116.023926 PMC5515592

[39] Zhang CX MicroRNAs: role in cardiovascular biology and disease. Clin. Sci, 2008, 114(12): 699–706. 10.1042/CS20070211 18474027

[40] Hinkel R; Ramanujam D; Kaczmarek V; AntimiR-21 prevents myocardial dysfunction in a pig model of ischemia/reperfusion injury. J. Am. Coll. Cardiol, 2020, 75(15): 1788–1800. 32299591 10.1016/j.jacc.2020.02.041

[41] Thum T; Gross C; Fiedler J; MicroRNA-21 contributes to myocardial disease by stimulating MAP kinase signalling in fibroblasts. Nature, 2008, 456(7224): 980–984. 19043405 10.1038/nature07511

[42] Yan MW; Chen C; Gong W; miR-21-3p regulates cardiac hypertrophic response by targeting histone deacetylase-8. Cardiovasc. Res, 2015, 105(3): 340–352. 25504627 10.1093/cvr/cvu254

[43] Patrick DM; Montgomery RL; Qi XX; Stress-dependent cardiac remodeling occurs in the absence of microRNA-21 in mice. J. Clin. Invest, 2010, 120(11): 3912–3916. 20978354 10.1172/JCI43604 PMC2964990

[44] Kamps JA; Krenning G Micromanaging cardiac regeneration: targeted delivery of microRNAs for cardiac repair and regeneration. World Journal of Cardiology, 2016, 8(2): 163–179. 26981212 10.4330/wjc.v8.i2.163 PMC4766267

[45] Van Rooij E; Purcell AL; Levin AA Developing microRNA therapeutics. Circ. Res, 2012, 110(3): 496–507. 22302756 10.1161/CIRCRESAHA.111.247916

[46] Huang CK; Bär C; Thum T miR-21, mediator, and potential therapeutic target in the cardiorenal syndrome. Front. Pharmacol, 2020, 11: 726. 32499708 10.3389/fphar.2020.00726 PMC7243366

[47] Guo JF; Song WP; Boulanger J; Dysregulated expression of microRNA-21 and disease-related genes in human patients and in a mouse model of alport syndrome. Hum. Gene Ther, 2019, 30(7): 865–881. 30808234 10.1089/hum.2018.205

[48] Ramanujam D; Schön AP; Beck C; MicroRNA-21-dependent macrophage-to-fibroblast signaling determines the cardiac response to pressure overload. Circulation, 2021, 143(15): 1513–1525. 33550817 10.1161/CIRCULATIONAHA.120.050682 PMC8032214

[49] Moghaddam AS; Afshari JT; Esmaeili SA; Cardioprotective microRNAs: lessons from stem cell-derived exosomal microRNAs to treat cardiovascular disease. Atherosclerosis, 2019, 285: 1–9. 30939341 10.1016/j.atherosclerosis.2019.03.016

[50] Song Y; Zhang C; Zhang JX; Localized injection of miRNA-21-enriched extracellular vesicles effectively restores cardiac function after myocardial infarction. Theranostics, 2019, 9(8): 2346–2360. 31149048 10.7150/thno.29945 PMC6531307

[51] Khatri N; Rathi M; Baradia D; *In vivo* delivery aspects of miRNA, shRNA and siRNA. Crit. Rev. Ther. Drug Carrier Syst, 2012, 29(6): 487–527. 23176057 10.1615/critrevtherdrugcarriersyst.v29.i6.20

[52] De Jong OG; Van Balkom BW; Schiffelers RM; Extracellular vesicles: potential roles in regenerative medicine. Front. Immunol, 2014, 5: 608.. 25520717 10.3389/fimmu.2014.00608 PMC4253973

[53] Johnson TK; Zhao LN; Zhu DH; Exosomes derived from induced vascular progenitor cells promote angiogenesis *in vitro* and in an *in vivo* rat hindlimb ischemia model. Am. J. Physiol.: Heart Circ. Physiol, 2019, 317 (4): H765–H776. 31418583 10.1152/ajpheart.00247.2019 PMC6843021

[54] Yin LY; Ohanyan V; Chilian WM; The role of MSC derived exosomes on cardiac microvascular dysfunction. Int. J. Cardiol, 2021, 344: 36–37. 34619264 10.1016/j.ijcard.2021.10.001

[55] Wang HY; Xie YL; Salvador AM; Exosomes: multifaceted messengers in atherosclerosis. Curr Atheroscler. Rep, 2020, 22(10): 57. 32772195 10.1007/s11883-020-00871-7

[56] Qiao L; Hu SQ; Liu SY; microRNA-21-5p dysregulation in exosomes derived from heart failure patients impairs regenerative potential. J. Clin. Invest, 2019, 129(6): 2237–2250. 31033484 10.1172/JCI123135 PMC6546482

[57] Luo QC; Guo DF; Liu GR; Exosomes from MiR-126-overexpressing ADSCs are therapeutic in relieving acute myocardial ischaemic injury. Cell Physiol Biochem., 2017, 44(6): 2105–2116. 29241208 10.1159/000485949

[58] Muthu S; Bapat A; Jain R; Exosomal therapy-a new frontier in regenerative medicine. Stem Cell Invest., 2021, 8: 7. 10.21037/sci-2020-037 PMC8100822 33969112

[59] Gray WD; French KM; Ghosh-Choudhary S; Identification of therapeutic covariant microRNA clusters in hypoxia-treated cardiac progenitor cell exosomes using systems biology. Circ. Res, 2015, 116(2): 255–263. 25344555 10.1161/CIRCRESAHA.116.304360 PMC4338016

[60] Sahoo S; Losordo DW Exosomes and cardiac repair after myocardial infarction. Circ. Res, 2014, 114(2): 333–344. 24436429 10.1161/CIRCRESAHA.114.300639

[61] Wang K; Jiang Z; Webster KA; Enhanced cardioprotection by human endometrium mesenchymal stem cells driven by exosomal MicroRNA-21. Stem Cells Transl Med., 2017, 6(1): 209–222. 28170197 10.5966/sctm.2015-0386 PMC5442741

[62] Bang C; Batkai S; Dangwal S; Cardiac fibroblast-derived microRNA passenger strand-enriched exosomes mediate cardiomyocyte hypertrophy. J. Clin. Invest, 2014, 124(5): 2136–2146. 24743145 10.1172/JCI70577 PMC4001534

[63] Small EM; Olson EN Peivasive roles of microRNAs in cardiovascular biology. Nature, 2011, 469(1330): 336–342. 21248840 10.1038/nature09783 PMC3073349

[64] Laggerbauer B; Engelhardt S MicroRNAs as therapeutic targets in cardiovascular disease. J. Clin. Invest, 2022, 132 (11): e159179. 35642640 10.1172/JCI159179 PMC9151703

